# Velocity Determinants in Spastic Patients after Stroke—A Gait Analysis Study

**DOI:** 10.3390/neurolint12030011

**Published:** 2020-11-06

**Authors:** Miguel Reis e Silva, Jorge Jacinto

**Affiliations:** Laboratório de Marcha, Centro de Medicina de Reabilitação de Alcoitão, 2649-506 Alcabideche, Portugal; jor.jacinto@netcabo.pt

**Keywords:** gait analysis, velocity, spasticity, stroke

## Abstract

Introduction: Gait velocity in spastic patients after stroke is both a life quality and mortality predictor. However, the precise biomechanical events that impair a faster velocity in this population are not defined. This study goal is to find out which are the gait parameters associated with a higher velocity in stroke patients with spastic paresis. Methods: The registries of a Gait analysis laboratory were retrospectively analyzed. The inclusion criteria were: trials of adult stroke patients with unilateral deficits. The exclusion criteria were: trials when patients used an external walking device, an orthosis, or support by a third person. Of the 116 initial patients, after the application of the exclusion criteria, 34 patients were included in the cohort, all with spatiotemporal, static and dynamic kinematic and dynamometric studies. Results: There was a correlation of velocity with cadence, stride length of the paretic (P) limb, stride length, and time of the P and non-paretic (NP) limb, double support time, all the parameters related to hip extension during stance phase, knee flexion during swing phase, and parameters related to ankle plantarflexion during stance phase. Conclusions: The main gait analysis outcomes that have a correlation with speed are related to the formula velocity = step length × cadence or are related to stance phase events that allow the anterior projection of the body. The only swing phase outcome that has a correlation with speed is knee flexion. More studies are needed from gait analysis laboratories in order to point out the most relevant goals to achieve with gait training in spastic stroke patients.

## 1. Introduction

Gait is an essential human function that allows us to freely move in our surrounding environment. Because of its implications, gait is provenly an important marker of quality of life [1] and even a mortality predictor [2], being already considered the sixth vital sign [3]. Stroke is one of the major causes of acquired disability [4]. It is estimated that 80% of stroke survivors have a walking disturbance [5]. Considering all this, improving walking velocity and efficiency for a stroke patient is an essential goal, in order to improve overall functionality and quality of life [6]. Gait velocity is the single most important characteristic, as it represents efficacy of this means of ambulation [7]. To be an autonomous community dweller, an individual must be able to perform at a minimum velocity (circa 0.7 m/s) [8]. Even if gait is an important goal in rehabilitation, there are only a few studies dedicated to finding the gait parameters that allow higher walking speeds; in fact, the only found reference dates from 1994, in which hip and ankle power during stance phase were a predictor a speed [9].

This study aims to find out which are the gait parameters associated with a higher velocity, in stroke patients with spastic paresis. It aims to provide new information regarding specific targets, towards which rehabilitation programs could be designed.

## 2. Methods

Patients records of a gait analysis laboratory were retrospectively analyzed. The inclusion criteria were: trials of adult stroke patients with unilateral deficits. The exclusion criteria were: trials when patients used an external walking device, an orthosis, or support by a third person. The retrospective analysis of the patient files was authorized by the Ethical Committee of Centro de Medicina de Reabilitação de Alcoitão (project identification number 14_2020) in 05/06/2020. The data collection didn’t involve any additional action beside the standard gait analysis exam and the personal information of the patients was blinded to the investigators so an informed consent wasn’t necessary.

The trials were captured using 6 infrared cameras Vicon T-series T10, 2 digital video cameras Basler piA1000-48gc, 4 force platforms AMTI OR6-7-2000, and 16 channel electromyography system Cometa Mini Wave Infinity.

A total of 116 patients were screened, with the inclusion of all the stroke patients admitted from 2013 to 2016. Of those, 15 were excluded for walking with the aid of an external individual and 67 were excluded for walking with an orthosis or walking device, resulting in 34 eligible subjects (Table 1).

Several outcomes were extracted from patients’ gait analysis records (Table 2); namely, all spatiotemporal parameters (automatically calculated by the devices), kinematics static parameters (calculated as averages of the short analyzed period), dynamic kinematics parameters (calculated manually by tracing the higher or lower value of the curves) and dynamometric parameters (also calculated manually as higher value of a curve). The type of initial contact of the paretic side during stance (forefoot vs. rearfoot) was subjectively analyzed on the video samples.

Statistical analysis was made using SPSS v.25 from IBM. Correlations of continuum variables were made using Pearson’s bivariate coefficient of the different variables and speed. Correlations with nominal variables as contact type were made using a *t*-test.

## 3. Results

The correlations between continuum variables and velocity are displayed in Table 3. Among the spaciotemporal parameters, there was a significant correlation of velocity with cadence, step and stride times, stride length of the non-paretic limb, and double support time. Regarding kinematic parameters, there was a significant correlation of velocity with the hip maximum extension during stance and unipodal stance phase, with hip excursion during unipodal stance phase, as well as with foot-off angle. In the knee, there was a significant correlation only with the maximum flexion during the swing phase. In the ankle, there was a significant correlation with the maximum plantar flexion during stance and unipodal stance phases, as well as its sagittal excursion during the unipodal stance phase. Regarding forces, there was a correlation between speed and the percentage of the gait cycle at which the first peak of vertical ground reaction force occurred, as well as with the maximum power produced at the ankle during stance phase.

There were 50% of the patients with forefoot initial contact (*n* = 17) and 50% with rearfoot initial contact (*n* = 17). The correlations of the variables with initial contact type are shown in Table 4. There was no statistical relation between initial contact type and velocity.

## 4. Discussion

### 4.1. Spatiotemporal Parameters

Velocity is the result of cadence multiplied by step/stride length, so it is not a surprise that cadence, double support time, and step time of both P and NP limbs are correlated with velocity. However, only stride length of the NP limb was associated with velocity, probably meaning that the compensation of the NP limb is more important than the deficit of the P limb.

### 4.2. Kinematic Parameters

In our cohort, none of the static kinematic parameters were correlated with velocity (we thought it was worth investigating, since these outcomes would be possible to evaluate even without instrumented gait analysis laboratories).

Regarding the hip extension, during stance phase was fundamental, with all hip extension-related outcomes showing significant correlations (more extension, higher velocity). Pelvic rotation is a known mechanism of compensation for the lack of hip extension during stance phase. On the other hand, maximum hip flexion during swing phase seems not to be relevant enough.

Regarding the knee, only maximum knee flexion angle during swing phase was correlated with velocity. This was the only joint with a significant correlation with velocity in which swing phase was concerned, suggesting an important role during this phase, since it is the joint with a higher sagittal range of motion during this phase of the gait cycle, in normal gait (hip 30°, knee 50°, ankle 10°).

Regarding the ankle, all the variables that correlated with velocity concerned late-stance plantarflexion, also known as push-off angle. The type of initial contact (forefoot vs. rearfoot) did not seem to have any repercussion on gait velocity.

### 4.3. Dynamometric Parameters

Consequent to the above-mentioned importance of the push-off, inferred from the kinematics outcomes of the ankle, the power produced at the ankle in pre-swing was also associated with velocity.

Moreover, the percentage of the gait cycle (stance phase) at which the first peak of the vertical ground reaction force occurred was also associated with velocity (the earlier the better), stressing the importance of the loading response and limb control to the efficacy of gait (velocity).

### 4.4. Integration of Concepts

**Figure 1:**
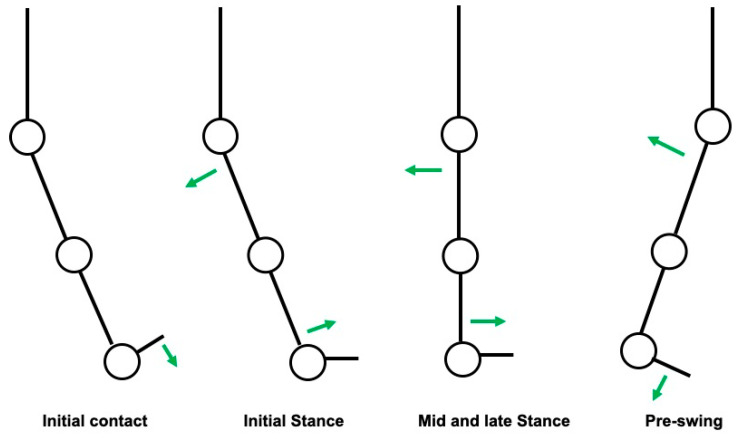
Schematization of the most relevant events during stance phase that are associated with higher gait velocity (green arrows: direction of force vectors).

During the loading response phase, immediately after the initial contact, there is a slight plantarflexion of the ankle in order to fully contact the ground. After this event, most of the stance phase is marked by a gradual extension of the hip with a concomitant passive dorsiflexion of the ankle in order to allow an anterior projection of the body. This seems to be the first, most important mechanism that has relevance for gait velocity. During pre-swing, there is an active plantarflexion of the ankle (push-off) that projects the body forward. This seems to be the second most important mechanism that has relevance for gait velocity. Both events are schematized in Figure 1.

During swing phase there is a triple flexion of the limb (hip and knee flexion with ankle dorsiflexion), that allows clearance in aerial limb progression. Only the knee flexion during this phase was correlated with velocity, which is in accordance with the fact that it is also the joint that has an larger sagittal range of motion during swing phase.

Taking into consideration the results, we hereby summarize the main parameters of gait, that have a correlation with velocity:

- Cadence and non-paretic stride length. The latter probably relates to the compensations accomplished by the healthy side of the body, possibly reflecting the muscle strength and motor control of the areas non affected by the stroke, or even the cardiorespiratory fitness of the subject (the capacity to compensate for the motor deficits is usually accompanied by an increased energy expenditure). The clinical implication of this finding is that the strength and motor control of the non-paretic limb may be a target for rehabilitation programs in order to better compensate for the deficits of the paretic limb and, consequently, allow for higher gait velocity.
- Maximum hip extension during the stance phase of the paretic limb: the hip is the main driving force responsible for the anterior projection of the body during early stance phase (the knee is in slight flexion in the early stance phase and the ankle in slight plantarflexion, followed by passive dorsiflexion, that compensates for hip extension). Considering this, the correlation found is actually plausible. However, slower gait patterns are usually associated with a shorter step length and, consequently, with a limited hip extension (there is not enough time for the hip to extend fully). Hence, a limited hip extension may be at the same time a cause and a consequence of slower gait. The clinical implication of this finding is that a spastic Iliopsoas muscle (sometimes overlooked in the clinical examination) should be carefully tracked.
- Knee flexion of the paretic limb during swing phase: the knee is the lower limb joint that shows a larger sagittal range of motion during swing phase; consequently, an impairment at this level would have a greater impact on aerial limb progression. The clinical implication of this finding is that spasticity of the Quadriceps muscle (which can contribute to a stiff-knee pattern in extreme situations) should be carefully tracked.
- Ankle plantar flexion of the paretic limb during pre-swing (which enables the push-off): The ankle is the main driving force responsible for the anterior projection of the body during the late stance phase, after the hip stops extending. The clinical implication of this finding is that the maintenance of Gastrocnemius and Soleus normal activation (timely) and muscle strength is relevant to gait velocity.

The strength of this study is in its relatively high number of participants with the same pathology, and that it relies on instrumented gait analysis data. The weakness may be in the outcomes that were chosen according to the clinical experience of the authors (there may be other relevant outcomes that were not analyzed).

We must point out that the described correlations do not specify if the outcomes associated with higher velocity are a cause (the outcome allows for higher velocity of gait) or a consequence (the outcome is a characteristic of gait at higher velocities).

## 5. Conclusions

Human gait is a complex sequence of events. There are two main characteristics of gait that are goals in rehabilitation programs: safety (falls prevention) and velocity (efficacy). Regarding the latter, to which this paper is dedicated, there seems to be a correlation of gait velocity with: (1) spatiotemporal parameters that are related to the formula velocity = step or stride length × cadence; (2) stance phase events that allow the anterior projection of the body; (3) knee flexion during swing phase. Further studies based on data from gait analysis laboratories are needed, in order to determine with more certainty the characteristics of gait that allow for higher velocity. This will point out the most relevant goals to achieve with gait training in spastic stroke patients, aiming to optimize custom rehabilitation programs.

## Figures and Tables

**Table 1:** Characterization of the analyzed sample.

**Number of Participants**	34
**Age**	49.7 (±13.4) years
**Sex**	32% (19) women/68% (15) men
**Paretic Limb**	62% (23) right/38% (11) left
**Speed**	0.5 (±0.2) m/s

**Table 2:** List of the registered outcomes (P—paretic; NP—non-paretic).

Spatiotemporal Parameters
Cadence
Double support time
Percentage of the gait cycle at which Foot off occurs
Limp index
Percentage of the gait cycle at which the opposite foot contacts the ground
Percentage of the gait cycle at which the opposite foot leaves the ground
Single support time
Step length
Step width
Step time
Stride length
Stride time
Walking speed
Initial contact type
Static kinematic parameters
Pelvic tilt
Hip angle (P and NP)
Knee angle (P and NP)
Ankle angle (P and NP)
Dynamic kinematic parameters related to paretic limb
Pelvic transverse plane excursion (rotation)
Pelvic maximum obliquity during swing phase
Hip—maximum flexion during swing phase
Hip—angle in initial foot contact
Hip—maximum extension during stance phase
Hip—maximum extension during unipodal stance phase
Hip—angle at foot-off
Hip—excursion during unipodal stance phase
Knee—maximum flexion during swing phase
Knee—initial contact angle
Knee—maximum extension during stance phase
Knee—angle at foot-off
Ankle—maximum dorsiflexion during swing phase
Ankle—initial contact angle
Ankle—maximum dorsiflexion during unipodal stance phase
Ankle—maximum plantar flexion during unipodal stance phase
Ankle—angle at foot-off
Ankle—excursion during unipodal stance phase
Force (vertical)—percentage of gait cycle of the first peak
Maximum potency of the ankle during stance phase

**Table 3:** Bivariate correlation coefficients (grey—statistically significant correlation: *p* < 0.05; * *p* < 0.05; ** *p* < 0.01).

Outcome	Pearson	Sig.
Age	0.155	0.382
Cadence	0.685 **	0.000
Double support time NP	−0.326	0.060
Foot-off NP	−0.445 **	0.008
Limp Index NP	0.148	0.405
Opposite Foot contact NP	−0.163	0.356
Opposite Foot off NP	−0.156	0.377
Single Support NP	−0.011	0.950
Step Length NP	0.166	0.349
Step Time NP	−0.543 **	0.001
Step width NP	−0.177	0.315
Stride Length NP	0.753 **	0.000
Stride Time NP	−0.651 **	0.000
Double support time	−0.414 *	0.015
Foot off P	0.056	0.754
Limp Index P	−0.177	0.317
Opposite Foot Contact P	−0.016	0.926
Opposite Foot Off P	−0.584	0.000
Single Support P	0.174	0.326
Step Length P	0.127	0.474
Step Time P	−0.550 **	0.001
Step width P	−0.021	0.905
Stride Length P	−0.125	0.481
Stride Time P	−0.665 **	0.000
Static kinematic—Pelvic Tilt	−0.191	0.278
Static kinematic—Hip angle NP	−0.122	0.491
Static kinematic—Hip angle P	−0.116	0.513
Static kinematic—Knee angle NP	−0.058	0.746
Static kinematic—Knee angle P	0.119	0.504
Static kinematic—Ankle NP	−0.028	0.874
Static kinematic—Ankle P	0.032	0.859
Pelvic amplitude of rotation	−0.200	0.256
Pelvic maximum obliquity during swing phase	0.099	0.585
Hip—maximum flexion during swing phase	0.100	0.572
Hip—angle at initial foot contact	0.221	0.210
Hip—maximum extension during stance phase	−0.447 **	0.008
Hip—maximum extension during unipodal stance phase	−0.431 **	0.011
Hip—Foot off angle	−0.626 **	0.000
Hip—excursion during unipodal stance phase	0.533 **	0.001
Knee—maximum flexion during swing phase	0.403 *	0.018
Knee—angle at initial contact	0.124	0.484
Knee—maximum extension during stance phase	0.154	0.384
Knee—angle at Foot-off	0.158	0.372
Ankle—maximum dorsiflexion during swing phase	0.112	0.528
Angle—angle at initial contact	0.009	0.958
Ankle—maximum plantarflexion during stance phase	0.359 *	0.037
Ankle—maximum dorsiflexion during unipodal stance phase	−0.152	0.392
Ankle—maximum plantarflexion during unipodal stance phase	0.371 *	0.031
Ankle—angle at foot-off	−0.299	0.085
Ankle—excursion during unipodal stance phase	0.641 **	0.000
Force (vertical)—percentage of gait cycle of the first peak	−0.620 **	0.000
Maximum potency of the ankle during stance phase	0.643 **	0.000

**Table 4:** *t*-test for correlation of velocity and initial contact type.

Initial Contact Type	t	df	Sig.
Velocity	−1.449	32	0.157
